# Improved Energy Storage Performance of Linear Dielectric Polymer Nanodielectrics with Polydopamine coated BN Nanosheets

**DOI:** 10.3390/polym10121349

**Published:** 2018-12-05

**Authors:** Jian Wang, Yunchuan Xie, Jingjing Liu, Zhicheng Zhang, Qiang Zhuang, Jie Kong

**Affiliations:** 1 Department of Materials Chemistry, School of Science, Xi’an Key Laboratory of Sustainable Energy Material Chemistry, Xi’an Jiaotong University, No. 28 Xianning West Road, Xi’an 710049, China; a1209113873@stu.xjtu.edu.cn (J.W.); jasmine1116@stu.xjtu.edu.cn (J.L.); zhichengzhang@mail.xjtu.edu.cn (Z.Z.); 2 Department of Applied Chemistry, School of Science, Northwestern Polytechnical University, No. 127 Youyi West Road, Xi’an 710072, China; zhuangqiang@nwpu.edu.cn (Q.Z.); kongjie@nwpu.edu.cn (J.K.)

**Keywords:** PVDF-based polymers, linear dielectric, polydopamine, BN nanosheets, energy density, discharging efficiency

## Abstract

Polymer-based nanodielectrics have been intensively investigated for their potential application as energy storage capacitors. However, their relatively low energy density (*U*_e_) and discharging efficiency (*η*) may greatly limit their practical usage. In present work, high insulating two-dimensional boron nitride nanosheets (BNNS), were introduced into a linear dielectric polymer (P(VDF-TrFE-CTFE)-*g*-PMMA) matrix to enhance the energy storage performance of the composite. Thanks to the surface coating of polydopamine (PDA) on BN nanosheets, the composite filled with 6 wt% coated BNNS (*m*BNNS) exhibits significantly improved breakdown strength (*E_b_*) of 540 MV/m and an energy density (*U*_e_) of 11 J/cm^3^, which are increased by 23% and 100%, respectively as compared with the composite filled with the same content of pristine BNNS. Meanwhile, *η* of both composites is well retained at around 70% even under a high voltage of 400 MV/m, which is superior to most of the reported composites. This work suggests that complexing polymer matrix with linear dielectric properties with surface coated BNNS fillers with high insulating 2D structure might be a facile strategy to achieve composite dielectrics with simultaneously high energy density and high discharging efficiency.

## 1. Introduction

Polymer-based film capacitors have been widely used for their fast charging and discharging speed, long cycling lifetime along with their low cost and facile processing performance. Theoretically, energy density (*U_e_*) stored in a dielectric material can be calculated from $U_{e}=\int_{0}^{D}EdD$, where *E* is the applied electric field, and *D* is the electric displacement of the dielectrics. For linear dielectric materials with consistent permittivity under varied electric fields, the above equation can be expressed as $U_{e}=1/2DE=1/2\varepsilon_{r}\varepsilon_{0}{\left(E_{b}\right)}^{2}$, where *ε_r_* and *ε*_0_ are permittivity of dielectrics and vacuum (8.85 × 10^−12^ F/m), respectively. Besides, the energy discharging efficiency (*η*) of the dielectrics could be defined as $\eta =U_{e}/\left(U_{e}+U_{l}\right)$, where *U_l_* is energy loss during charging-discharging cycles. Apparently, dielectric materials with large dielectric permittivity (*ε_r_*), high breakdown strength (*E_b_*), and low *U_l_* are preferred to realize capacitors featuring as high *U_e_* and high *η* [1,2,3,4,5,6].

Polymer/ceramic nanodielectrics, which may combine merits of high *E_b_* of the polymer matrix and high *ε_r_* of the inorganic particles, have attracted considerable research interests during past decades for their wide range of application as high-performance energy storage capacitors in modern smart grids, new energy vehicles, and high-power weapons [7,8,9,10,11,12]. Although inspiring progress has been achieved, great challenges for this kind of composite still remain from the practical point of view. On the one hand, poly(vinylidene fluoride) (PVDF) based polymers are usually chosen as the matrix for its high and tunable *ε_r_* value (10–100). However, their bulk hysteresis loss is as high as 40–60% due to dipolar ferroelectric relaxations, which resulted in high energy loss and seriously limit their practical applications [13,14,15]. On the other hand, BaTiO_3_ based ferroelectric ceramics are generally used as fillers for their high *ε_r_* values (~10^3^). Unfortunately, high volume filler loadings (>30 vol%) are needed to obtain composites with high *ε_r_* based on series or parallel model, which leads to dielectrics with increased conductive loss, significantly reduced *E_b_*, thus, low *U_e_* and high energy loss [16,17,18]. This problem can be obviously alleviated by constructing core-shell structured particles or utilizing high aspect ratio particles. Recently, the two-dimensional nanosheets, such as boron nitride and mica nanosheets with excellent insulating and heat conducting properties, have been well reported to blend with polymers to achieve high *E_b_* and high *η* at the same time [19,20,21].

Inspired by the above ideas, a linear dielectric polymer, grafted terpolymer P(VDF-TrFE-CTFE)-*g*-PMMA containing 20 wt% PMMA, is utilized as the matrix for its typical linear dielectric character under increased electric field with rather low *U_l_* herein [22,23]. In addition, polydopamine coated boron nitride nanosheets (*m*BNNS) with excellent electrical properties, including *ε_r_* of ~4, dielectric loss (tanδ) of 0.001–0.008, resistivity of ~10^14^ Ω/cm and *E_b_* of ~380 MV/m [24,25], are added into the polymer matrix to achieve composites with simultaneously high *E_b_* and low *U_l_* via a solution-cast process. Thanks to the enhanced compatibility of the two phases by polydopamine (PDA) layer and barrier effect of high insulating 2D fillers, the optimized *m*BNNS filled composite presents the promising *E_b_* of 540 MV/m and *U_e_* of 11.0 J/cm^3^, which is nearly 123% and 200% that of the boron nitride nanosheets (BNNS) filled composite, respectively. Most interestingly, a high *η* of 70% is retained at an electric field up to 400 MV/m in the composites, which is much larger than most of the reported results.

## 2. Experimental

### 2.1. Materials

Poly(vinylidene fluoride-chlorotrifluoroethylene) (P(VDF-CTFE), VDF/CTFE = 78/22 in molar ratio) was purchased from Zhonghao Chenguang Research Institute of Chemical Industry (Chengdu, China). *h*-BN powder was obtained from Nanjing XFNANO Materials Tech. Co. Ltd. (99.99%, Nanjing, China) *N*-methyl pyrrolidone (NMP), *N*,*N*-dimethyl formamide (DMF) and anhydrous ethanol were bought from Tianjin Chemical Reagents Co. Ltd. (AR grade, Tianjin, China). Methyl methacrylate (MMA) (99%, Aladdin Reagents Co. Ltd., Shanghai, China) was washed with 5 wt% NaOH solution and distilled under reduced pressure to remove the inhibitor before use. CuCl (97%), Cu powder (75 μm, 99%) from Sinopharm Reagents Co. Ltd. (Beijing, China), and 2,2-bipyridine (BPy, 99%) from Alfa Aesar Reagents Co. Ltd. (Haverhill, MA, USA) were used as received.

### 2.2. Synthesis of Liner Ferroelectric P(VDF-TrFE-CTFE)-g-PMMA

The synthesis of Poly(vinylidene fluoride-trifluoroethylene-chlorotrifluoroethylene) (P(VDF-TrFE-CTFE)) with a molar ratio of VDF/TrFE/CTFE = 78/16/6 follows the hydrogenation process of P(VDF-CTFE) as reported in the previous work [26]. The synthesis of P(VDF-TrFE-CTFE)-*g*-PMMA follows the grafting reaction of methyl methacrylate (MMA) from P(VDF-TrFE-CTFE) using ARGET-ATRP (activators regenerated by electron transfer for atom transfer radical polymerization) as reported in our previous work [27,28]. Grafted terpolymers (P(VDF-TrFE-CTFE)-*g*-PMMA) bearing varied PMMA concentrations are obtained by altering either the reaction time or the monomer concentration in the feed during each reaction. Specifically, the P(VDF-TrFE-CTFE)-*g*-PMMA containing 20 wt% PMMA side chains is chosen as the polymer matrix in this work for its excellent linear dielectric character. The chemical structure and dielectric properties of the polymer matrix are characterized via ^1^H-NMR, FTIR and *D-E* loops measurements as shown in Figures S1 and S2. The dielectric constant and energy storage properties of the P(VDF-TrFE-CTFE) and P(VDF-TrFE-CTFE)-*g*-PMMA matrix are presented in Figure S3.

### 2.3. Preparation of Exfoliated BN Nanosheets and Polydopamine Coated BN Nanosheets

BN nanosheets, marked as BNNS, were physically exfoliated from *h*-BN powder in DMF following a sonication-centrifugation process [29]. Generally, 0.5 g *h*-BN powder was dispersed in 100 mL DMF under vigorous stirring followed by tip-type sonication (800 W, JY96-IIN, Ningbo Scientz, China) for 6 h (320 W, 800 W × 40%). The resultant mixture was firstly centrifuged at 1000 rpm for 30 min, and the collected supernatant was subjected to a 10 min centrifugation at 10,000 rpm. BNNS were obtained after drying the precipitate overnight at 75 °C under reduced vacuum.

PDA coated BNNS, marked as *m*BNNS, was prepared with the process described in reference [30]. Generally, 0.2 g BNNS were well dispersed into 50 mL deionized water through ultrasonic treatment (100 W) for 30 min. Sixty-seven thousands of a gram of dopamine and 150 mL Tris buffer were added into above suspension followed by vigorous stirring at room temperature in air for 48 h. *m*BNNS were collected by centrifuging the suspension at 5000 rpm for 5 min followed by washing the sediment with anhydrous ethanol for five times and drying the sediment at 80 °C for 12 h.

### 2.4. Fabrication of Nanodielectrics

**Figure 1:**
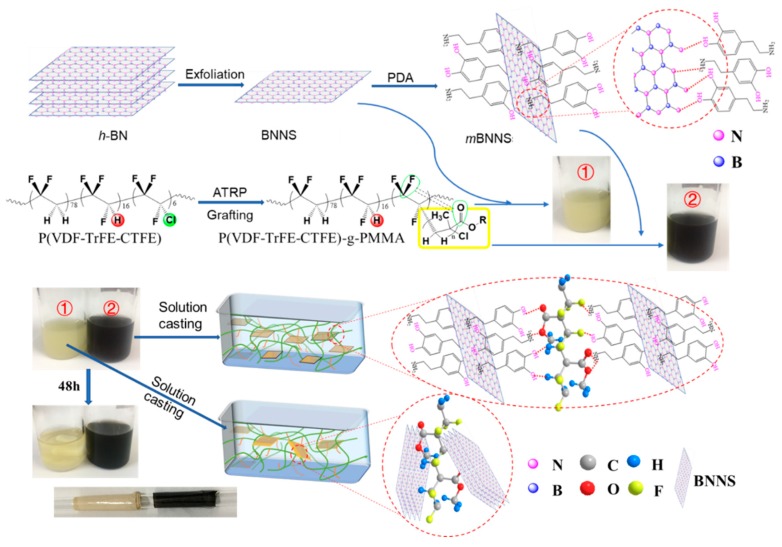
Schematic illustration for preparation of polymer nanocomposite film.

A typical solution casting process was conducted to fabricate nanodielectric films as illustrated in Figure 1 [31,32]. Firstly, BNNS and *m*BNNS were dispersed into DMF, the obtained suspension was ultrasonicated and vigorously stirred for 30 min before mixing with the grafted terpolymer solution (6 wt% solution concentration) in DMF. The grafted terpolymer P(VDF-TrFE-CTFE)-*g*-PMMA containing 20 wt% PMMA side chain was chosen as the polymer matrix to fabricate composite dielectrics for its excellent dielectric properties. The composite films were fabricated by cast the suspensions bearing BNNS or *m*BNNS fillers of various weight contents and polymer matrix onto glass slides, followed by removing the volatiles completely at 75 °C for 4 h.

Two series of composite films containing predefined weight fractions of BNNS or *m*BNNS (0 wt%, 2 wt%, 4 wt%, 6 wt%, 8 wt%, and 10 wt%), including P(VDF-TrFE-CTFE)-*g*-PMMA/BNNSs and P(VDF-TrFE-CTFE)-*g*-PMMA/*m*BNNSs, were prepared under the above conditions, respectively. The resultant composite films with a thickness of ~20 μm were peeled off from the slides and sputtered with Au on both surfaces as electrodes for subsequent electrical measurements. As illustrated in Figure 1, *m*BNNS particles exhibit much better dispersion in DMF than neat BNNS, which benefits the following fabrication process of *m*BNNS filled composites. Nanocomposite films, thus, prepared had good flexibility and could be rolled on the glass rod as shown in inset picture of Figure 1.

### 2.5. Characterization

Transmission electron microscopy (TEM) observation was operated on a JEM-200CX (JEOL Ltd., Tokyo, Japan)by placing a few drops of the dispersion (BN particle in ethanol) on a copper grid, and evaporating ethanol at room temperature before observation. Field-emission scanning electron microscopy (FE-SEM) results were obtained on Hitachi SU8020 (Hitachi Ltd., Tokyo, Japan), and the samples were fractured with liquid nitrogen and coated with a thin layer of gold before observation. Atomic force microscope (AFM) measurements were conducted on a Veeco-8 (Bruker Inc., Billerica, MA, USA) equipped with a tapping probe, and the samples were prepared by drop casting the dispersion (particle in ethanol) onto a clean Si substrate.

Fourier-transform infrared (FT-IR) spectra were observed with an IR spectrophotometer (Bruker Inc., Karlsruhe, Germany) with a resolution of 2 cm^−1^ and 16 scanning times. For inorganic particles, 1 wt% particle was ground with KBr powder and then pressed into circular samples under 5 MPa. X-ray diffraction (XRD) measurement was conducted on a Bruker D8 ADVANCE diffractometer (Bruker Inc., Karlsruhe, Germany) with the X-ray wavelength of 1.542 Å (Cu Kα radiation, 40 kV and 100 mA), the 2*θ* diffraction angle from 20° to 80°, the rate of 15°/min and the step of 0.02°. Proton nuclear magnetic resonance (^1^H-NMR) spectra were carried out on a Bruker (Advance III) 400 MHz spectrometer (Bruker Inc., Karlsruhe, Germany) with acetone-d_6_ as solvent and tetramethylsilane as an internal standard.

The dielectric properties of all composites were obtained via a Novocontrol Concept 80 (Novocontrol Technologies GmbH & Co. KG, Montabaur, Germany ) at frequencies ranging from 1 Hz to 1 MHz with 1 V voltage at room temperature. High-field *D-E* loops were collected using a Sawyer-Tower circuit, where the samples were subjected to a triangular unipolar wave with a frequency of 10 Hz. Electric breakdown strength results were obtained on an auto voltage withstanding tester (RK2674B) under a direct-stress voltage ramp of 500 V/s in accordance with the ASTM D149 standard. Gold electrodes of diameter 10 mm and thickness around 30 nm were sputtered on both sides of the composite films by JEOL JFC-1600 auto fine coater (JEOL Ltd., Tokyo, Japan) for all electric properties characterizations.

## 3. Results and Discussion

### 3.1. Chemical Structure and Morphology of BNNS and mBNNS

**Figure 2:**
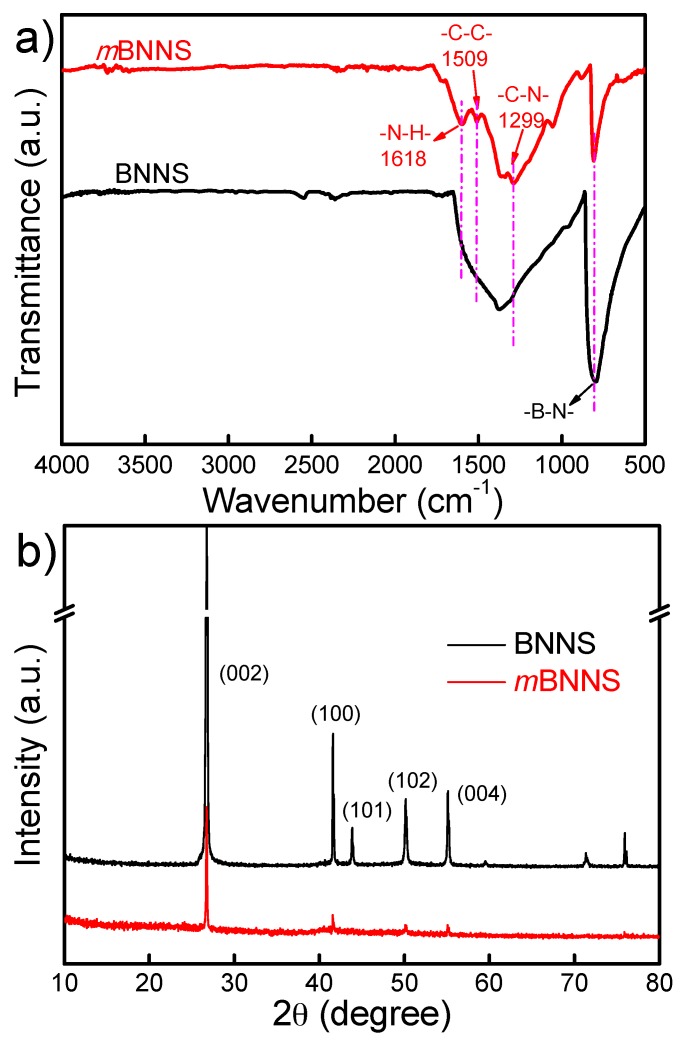
(**a**) Fourier-transform infrared (FT-IR) spectra and (**b**) X-ray diffraction (XRD) patterns for boron nitride nanosheets (BNNS) and *m*BNNS particles, respectively.

The FT-IR results of BNNS and *m*BNNS particles are compared, and the results are shown in Figure 2a. The new absorption bands appeared at 1618 cm^−1^ and 1299 cm^−1^ in *m*BNNS are mainly attributed to the bending vibration of N–H bond and C–N bond stretching vibration in the aromatic amine, respectively. The stretching vibration of aromatic C–C bond could be confirmed by the absorption peak at 1509 cm^−1^, which suggests the successful coating of PDA molecules onto BNNS particle surface [33,34].

Figure 2b presents the XRD patterns of neat BNNS and *m*BNNS particles, respectively. Apparently, all the characteristic diffraction peaks can be well assigned to the crystal form of *h*-BN, such as peaks at 26.8° (002), 41.6° (100), 50.1° (102), and 55.1° (004), which fits well with the standard cards (JCPDS 73-2095) [35]. Coating BNNS with PDA does not change the crystal form of the BNNS particle.

**Figure 3:**
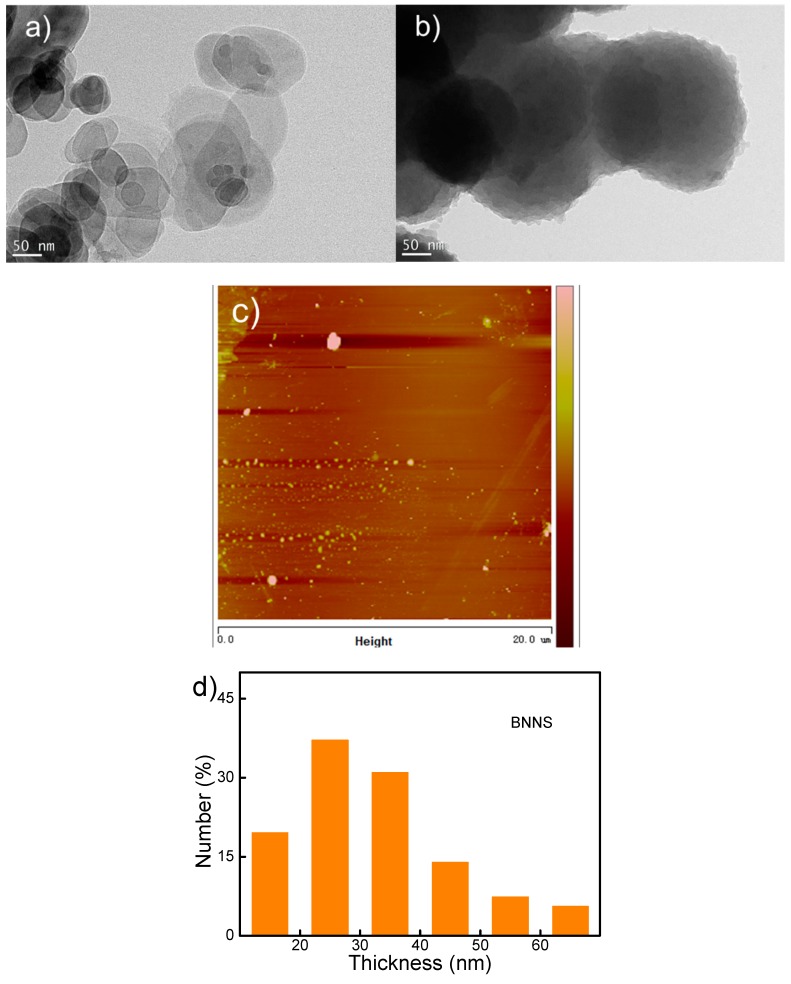
Transmission electron microscopy (TEM) images for (**a**) BNNS and (**b**) *m*BNNS particles, respectively, (**c**) AFM image and (**d**) the histogram for BNNS.

Figure 3a,b show the typical TEM images of BNNS particles before and after PDA coating, respectively. Clearly, neat BNNS particles possess few layered structures with smooth edges. After coated with PDA, *m*BNNS particles present blurred edge suggesting complete PDA encapsulation around its surfaces. To assess the thickness of BNNS particles, AFM imaging is performed at multiple locations across the sample. The absolute thickness of BNNS particles is measured, and its thickness distribution histogram is plotted in Figure 3d. The thickness of BNNS particles ranges from 10–70 nm region with a distribution peak at ~25 nm.

### 3.2. Chemical Structure and Morphology of the Composites

**Figure 4:**
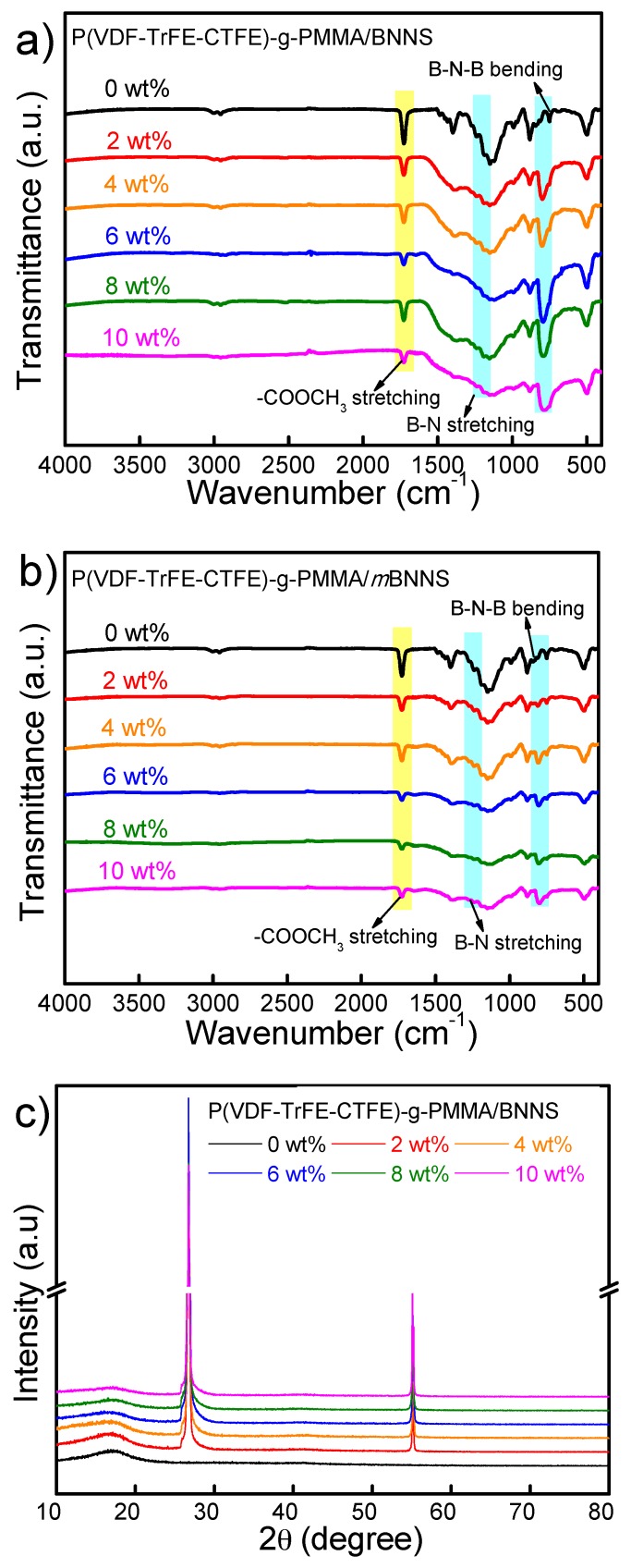

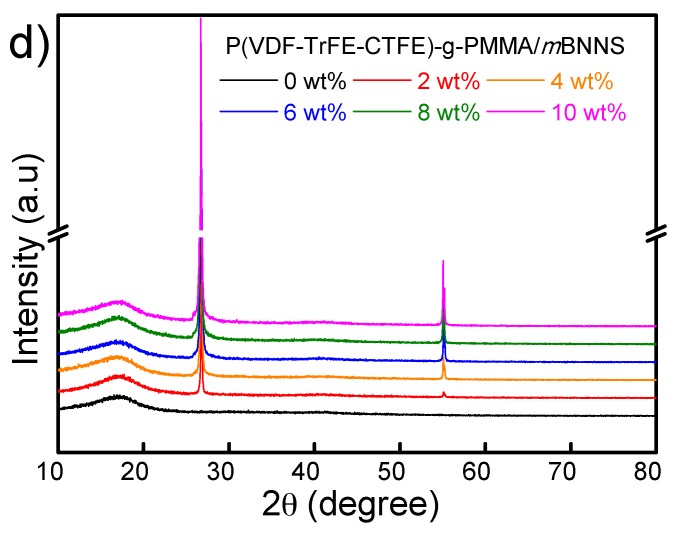
(**a**,**b**) FT-IR spectra and (**c**,**d**) XRD patterns for the composites containing varied BNNS and *m*BNNS particles, respectively.

The chemical composition and morphology of the composites are characterized by FT-IR, XRD, and SEM. The FT-IR spectra of two series of composites, including P(VDF-TrFE-CTFE)-*g*-PMMA/BNNSs and P(VDF-TrFE-CTFE)-*g*-PMMA/*m*BNNSs, are shown in Figure 4a,b, respectively. A comparison with the matrix reveals no particular new absorption peaks in the two sets of composites profiles, meaning that the introduction of BNNS or *m*BNNS fillers does not alter the chain conformation or crystal form of the matrix. Figure 4c,d present the XRD spectra of the two set of composites, respectively. The grafted terpolymer matrix shows an amorphous structure with invisible diffraction peak except for a wide and dispersed peak at about 18.1°. With the increasing content of BNNS or *m*BNNS (>4 wt%), the sharp diffraction peaks of the fillers (26.8°, 55.1°) dominate the XRD profile and their diffracting intensities are gradually increased.

**Figure 5:**
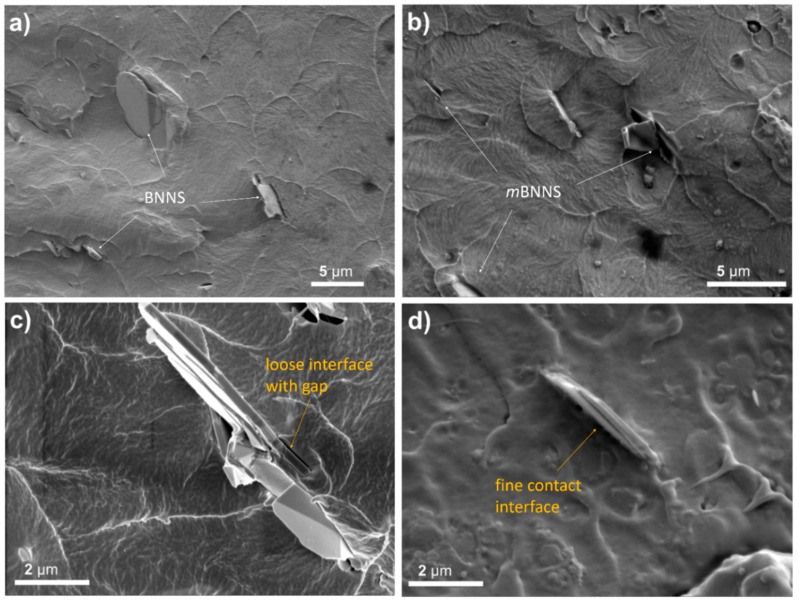
Cross-sectional scanning electron microscopy (SEM) images of the composite films containing 6 wt% (**a**,**c**) BNNS and (**b**,**d**) *m*BNNS particles.

The cross-sectional morphology of two series of composites is observed through SEM, as presented in Figure 5. Comparing to BNNS filled composite, *m*BNNS filled composites show rough surface morphology with more ravine-like structures. That suggests typical ductile fractures and strong interfacial interactions of the polymer matrix and the filler after PDA coating. Meanwhile, unlike the distinct and loose interface of BNNS particles with the matrix, the interface between *m*BNNS particles and the matrix is finely contacted and rather blurry. That may be assigned to enhanced interactions between the two phases, which is induced by hydrogen bonds, formed by –NH–, –OH groups of PDA molecules and –C–F dipoles of the matrix, and static-electronic attractions between the dipoles of two phases.

### 3.3. Dielectric Properties of the Composites

**Figure 6:**
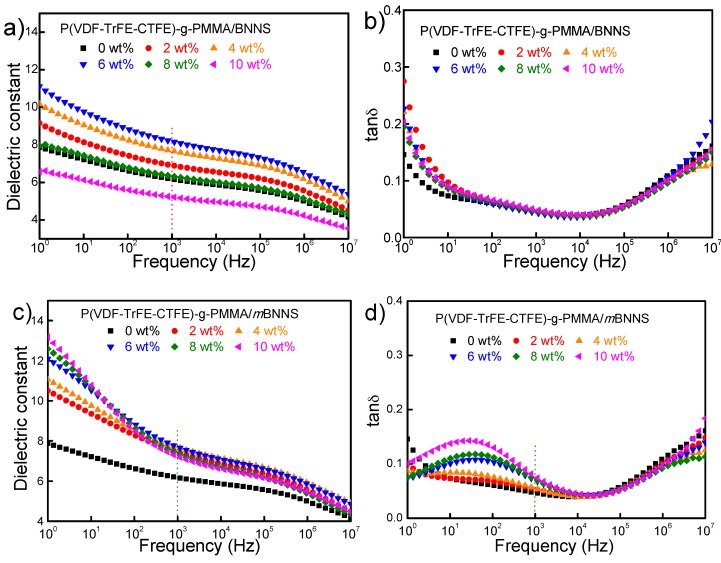
Frequency dependence of (**a**,**c**) dielectric constant and (**b**,**d**) dielectric loss of the composites with different particle contents under room temperature.

The frequency dependence of the dielectric constant of two series of nanocomposites measured on a Novocontrol C80 instrument at room temperature is shown in Figure 6a,c. The dielectric constant of both composites is gradually reduced as a function of frequency, which is rather similar to the changing tendency of the neat polymer matrix. For BNNS filled composites, the dielectric constant increases first with the increasing of filler content and reaches a peak value of ~11@100Hz when the filler content reaches 6 wt%. It starts to decrease with the further increase of loading content. That was caused by the introduction of BNNS particles, which might insert between macromolecules and resulted in reduced chain disentanglements and, thus, easier dipoles orientation. However, as the filler content beyond 6 wt%, too much BNNS with low dielectric constant (~4) may obviously depress the overall polarization. For *m*BNNS filled composites, their dielectric constant increases with the increased filler contents and reaches a peak value of ~13 at 10 wt% level. Obviously, after PDA coating, strong dipoles including –NH– and –OH around coated particles may strengthen the interfacial polarization of the composite thus leading to increased dielectric constants.

It is well accepted that dielectric losses in polymer materials at a lower frequency (<10^2^ Hz) mainly originates from ion conduction, and dielectric response hysteresis of dipoles is mainly responsible for the losses at a higher frequency (~10^6^ Hz) [36]. The frequency dependence of dielectric loss (or tanδ) of two series of composites is plotted in Figure 6b,d. Two competing effects for the introduction of 2D fillers may exist. On the one hand, the introducing of inorganic particles into the polymer matrix may enhance the interfacial polarization. On the other hand, the high insulating 2D filler may help to reduce the ion conduction. That could explain the changing tendency of tanδ for BNNS filled composites. For *m*BNNS filled composites, there presents a relaxation peak at ~ 100 Hz relating to interfacial polarization. That should be mainly caused by the PDA layer containing –NH– and –OH groups that could easily form hydrogen bonds with the matrix and, thus, increased interfacial polarization as reported [33]. At higher frequency (~10^6^ Hz), no significant difference in losses between the two composites could be found, suggesting that the introduction of particles resulted in a negligible influence on the dipole relaxation of the terpolymer matrix.

### 3.4. Electrical Breakdown of the Composites

**Figure 7:**
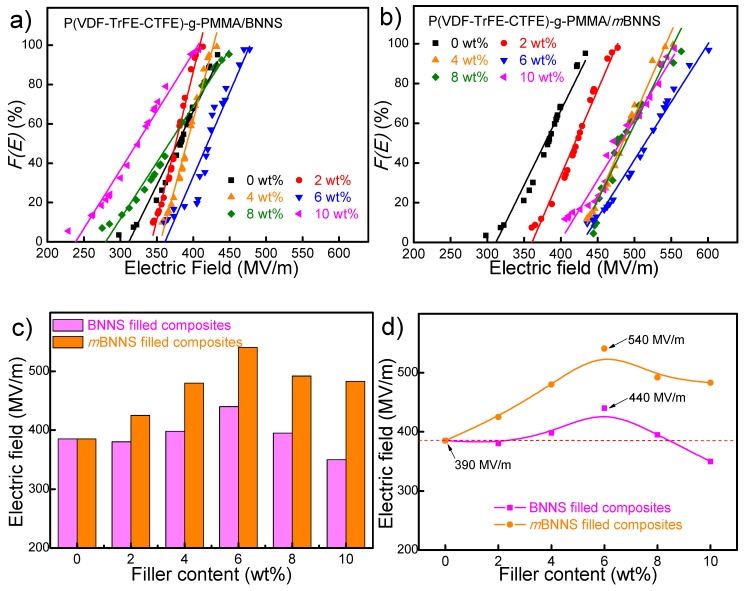
Weibull distribution of breakdown strength for the two sets of composites with varied (**a**) BNNS, (**b**) *m*BNNS contents, and (**c**,**d**) breakdown strength vs. filler contents of the composites under room temperature.

As indicated in the equation ($U_{e}=1/2\varepsilon_{r}\varepsilon_{0}{\left(E_{b}\right)}^{2}$) [37], elevated breakdown strength of materials is rather important for achieving superior energy density. A two-parameter Weibull statistic is used to analyze *E_b_* values for both series of composites as described in Equation (1) [38],

(1) $$F\left(E\right)=1-\exp\left[-{\left(\frac{E}{\alpha }\right)}^{\beta }\right]$$

where *F*(*E*) is the cumulative probability of electric failure, *E* is the measured breakdown field, the scale parameter *α* is the field strength for which there is a 63% probability for the sample to break down, and the shape parameter *β* evaluates the scatter of data and a higher value of *β* represents greater dielectric reliability. As shown in Figure 7a–c, the maximum breakdown strength of BNNS filled composites and *m*BNNS filled composites is observed as 440 MV/m and 540 MV/m at 6 wt% filler content, respectively, and a further increase in filler content leads to a gradual decrease in *E_b_*. Unlike BNNS filled composites, it could be noted that all *E_b_* values of *m*BNNS filled composites are larger than that of polymer matrix (385 MV/m) within the whole range of filler loadings as shown in Figure 7d. That could be ascribed to the enhanced compatibility and interfacial interactions between the two phases as discussed above. Apparently, *m*BNNS filled composites possess better interfacial interactions with the matrix, which could weaken the uneven distribution of the electric field inside the composites and improve the breakdown strength.

### 3.5. Energy Density and Discharging Efficiency of the Composites

**Figure 8:**
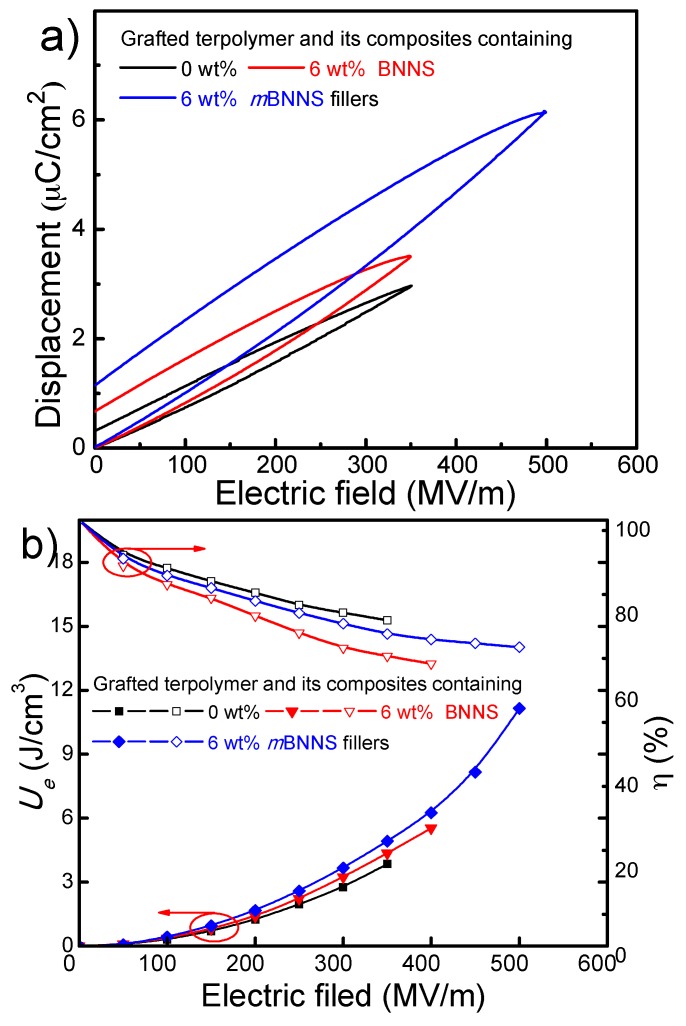
(**a**,**b**) Unipolar *D-E* hysteresis loops, (**c**,**d**) *U_e_* and *η* of the two composites containing 6 wt% BNNS and *m*BNNS particles, respectively.

The energy storage capacity of the dielectric materials was evaluated using unipolar *D-E* loops under increased electric fields, where neat P(VDF-TrFE-CTFE)-*g*-PMMA matrix, BNNS, and *m*BNNS filled composites with 6 wt% particle content are considered as instances (Figure 8). It can be observed that *m*BNNS filled composite has the maximum electric displacements as high as ~6.2 μC/cm^2^, which is nearly 100% higher than that of BNNS filled composite (Figure S4). Meanwhile, the breakdown strength of *m*BNNS filled composite is estimated to be ~500 MV/m, also larger than those of BNNS filled composite and the matrix. That agrees well with *E_b_* values obtained using the Weibull distribution model.

As shown in Figure 8b, the largest discharged *U_e_* derived from unipolar *D-E* loops of *m*BNNS filled composite is nearly 11.0 J/cm^3^, which is about three times and two times as those obtained from grafted terpolymer P(VDF-TrFE-CTFE-*g*-PMMA) matrix (~3.8 J/cm^3^) and BNNS filled composite (~5.5 J/cm^3^) at breakdown strength, respectively. The greatly enhanced *U_e_* may be contributed to by simultaneously increased *ε_r_* and *E_b_* values for the composite. Meanwhile, it is interesting that *η* values of the two composites containing 6 wt% filler content are estimated to be ~70%@400 MV/m, which is larger than most of the reported work (40–60%) [39,40]. This could be explained by the significantly reduced conduction loss of the composite induced by the improved compatibility between the grafted terpolymer matrix and PDA coated particles.

## 4. Conclusions

In this work, nanodielectrics with simultaneously high energy density and discharging efficiency were fabricated by using a linear dielectric matrix with low hysteresis loss and polydopamine coated BNNS with a high insulating property. The results showed that the PDA layer formed around BNNS particles could effectively improve the interfacial compatibility and facilitate the formation of strong interactions between the two phases, which resulted in enhanced dielectric permittivity and breakdown strength and, thus, high energy density of 11.0 J/cm^3^ for the composite. Besides, thanks to the linear polymer matrix and high insulating fillers with 2D structure, *m*BNNS filled composite films with good flexibility presented enhanced discharging efficiency of nearly 72%, which is superior to most of the reported values. This work might offer a facile way for the scalable fabrication of energy storage dielectrics with simultaneously enhanced energy density and discharging efficiency.

## Supplementary Materials

The following are available online at http://www.mdpi.com/2073-4360/10/12/1349/s1.
