# Supplementary material for: Improved Energy Storage Performance of Linear Dielectric Polymer Nanodielectrics with Polydopamine coated BN Nanosheets
Source: Polymers (Basel). 2018 Dec 5;10(12):1349. doi: 10.3390/polym10121349 (PMC6401857; doi:10.3390/polym10121349)
Supplement: Supplementary file 1 [file polymers-10-01349-s001.pdf]

Supporting Information

Improved Energy Storage Performance of Linear Ferroelectric Polymer Nanodielectrics with Polydopamine coated BN Nanosheets

Jian Wang[a], Yunchuan Xie[a*], Jingjing Liu[a], Zhicheng Zhang[a], Qiang Zhuang[b] and Jie Kong[b]

[a] Department of Materials Chemistry, School of Science, Xi'an Key Laboratory of Sustainable Energy Materials Chemistry, Xi'an Jiaotong University, No. 28 Xianning West Road, Xi'an 710049, China;

[b] Department of Applied Chemistry, School of Science, Northwestern Polytechnical University, No. 127 Youyi West Road, Xi'an 710072, China;

a1209113873@stu.xjtu.edu.cn (J. Wang); jasmine1116@stu.xjtu.edu.cn (J. Liu); zhichengzhang@mail.xjtu.edu.cn (Z. Zhang); zhuangqiang@nwpu.edu.cn (Q. Zhuang); kongjie@nwpu.edu.cn (J. Kong)

[*] Correspondence: ycxie@xjtu.edu.cn; Tel.: +86-29-8266-3914

Chemical structure of grafted terpolymer P(VDF-TrFE-CTFE)-g-PMMA

Successful grafting of PMMA side chains from Cl sites in P(VDF-TrFE-CTFE) is characterized with ^{1}H NMR as shown in **Fig. S1**a. Comparing with P(VDF-TrFE-CTFE), new signals at 3.6-3.7 ppm is assigned to the protons on *-OCH3* of MMA in PMMA grafted terpolymers. The grafting of PMMA onto P(VDF-TrFE-CTFE) could also be confirmed by FTIR measurement as shown in **Fig. S2**b. The absorption band at 1726 cm^{-1} is assigned to the C=O on acrylate ester for the grafted terpolymer.

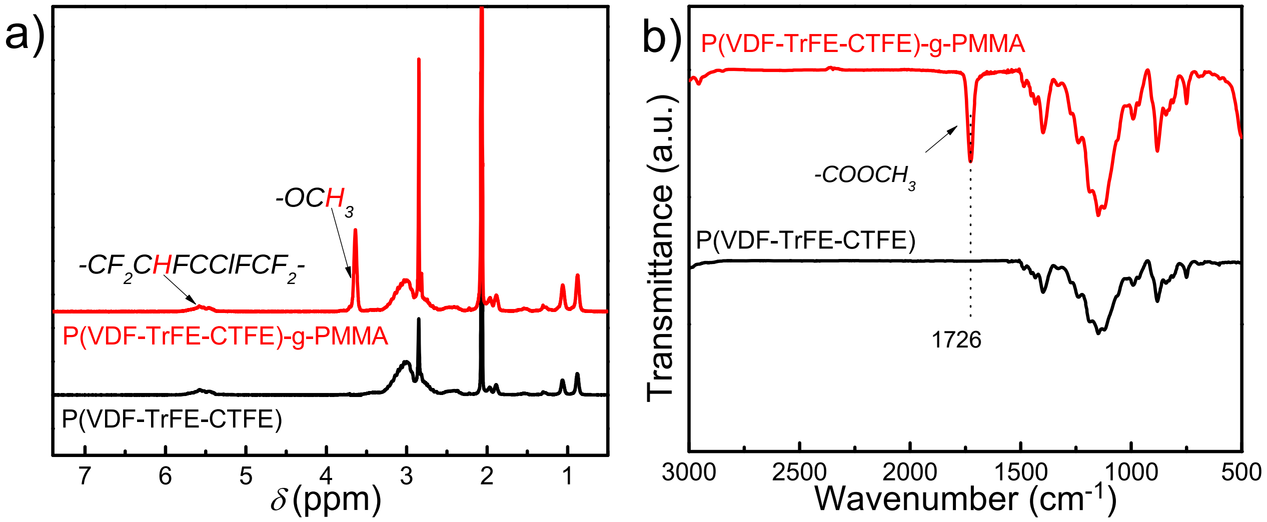

Fig. S1 (a) ^{1}H-NMR and (b) FT-IR spectra of P(VDF-TrFE-CTFE) and P(VDF-TrFE-CTFE)-g-PMMA containing varied PMMA concentration.

Chemical structure of grafted terpolymer P(VDF-TrFE-CTFE)-g-PMMA

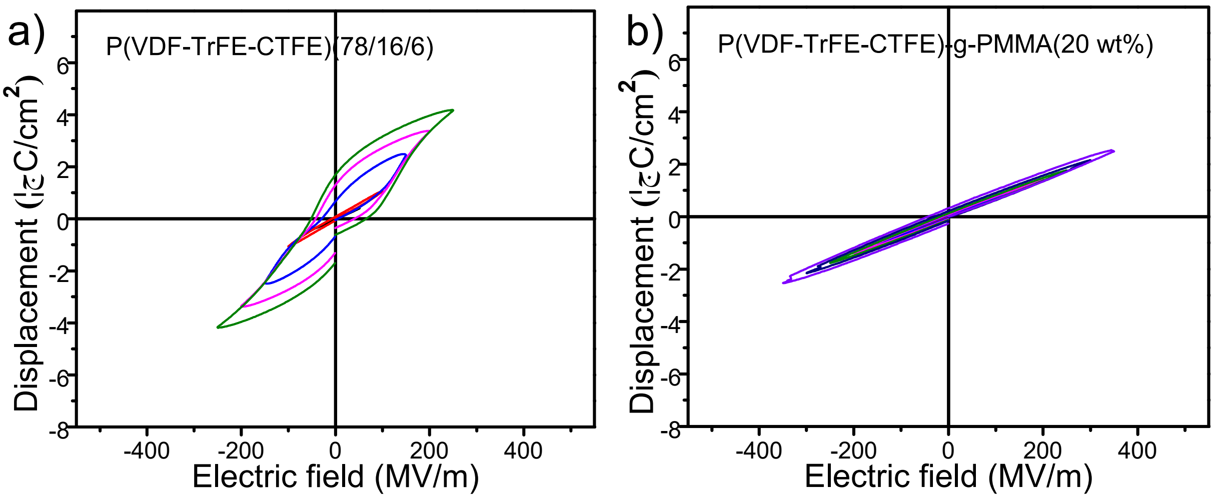

Fig. S2 Unipolar *D-E* hysteresis loops of P(VDF-TrFE-CTFE) (a) and P(VDF-TrFE-CTFE)-g-PMMA (b) containing 20 wt% PMMA, respectively.

Dielectric properties of the polymers and the composites

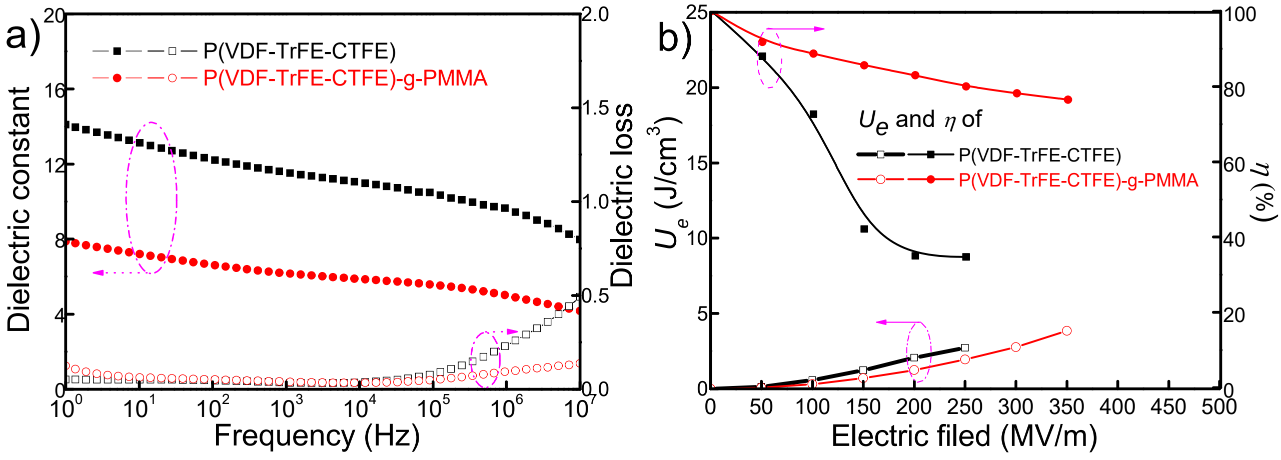

Fig. S3 (a) Dielectric constant and dielectric loss, (b) U_e and η of P(VDF-TrFE-CTFE) and P(VDF-TrFE-CTFE)-g-PMMA, respectively.

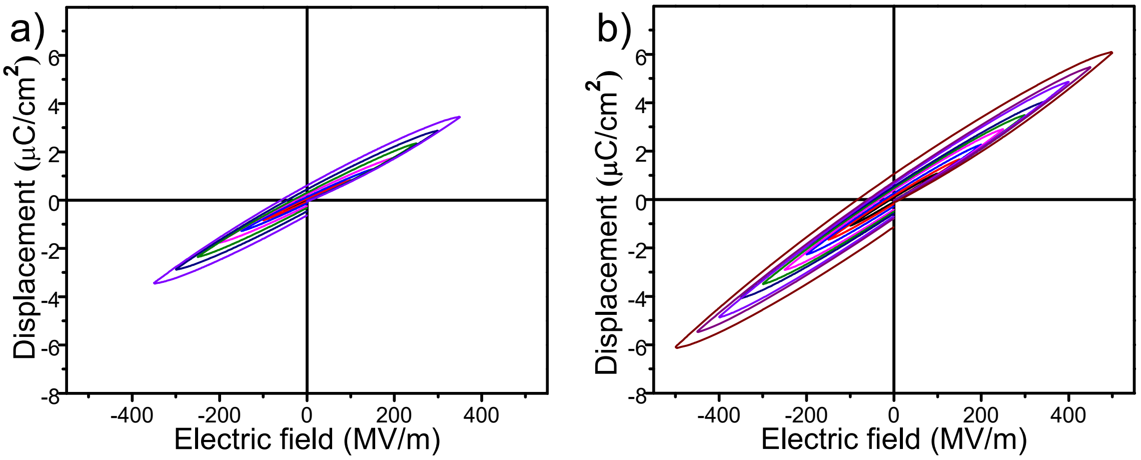

Fig. S4 Dipolar *D-E* hysteresis loops of (a) P(VDF-TrFE-CTFE)-g-PMMA/BNNS and (b) P(VDF-TrFE-CTFE)-g-PMMA/*m*BNNS composites with 6 wt% filler content.
